# The Bactericide Effects of Chitosan When Used as an Indicator of Chlorine Demand

**DOI:** 10.3390/polym17091226

**Published:** 2025-04-30

**Authors:** Josefine Molina-Pinna, Félix R. Román-Velázquez

**Affiliations:** Department of Chemistry, University of Puerto Rico, Mayagüez Campus, Mayagüez, PR 00681, USA; felixr.roman@upr.edu

**Keywords:** chitosan biopolymer, water treatment plant, high raw water turbidity, chitosan bactericide effect, chlorine demand, sodium hypochlorite, breakpoint

## Abstract

The Miradero Water Treatment Plant (MWTP) in Mayagüez, Puerto Rico, uses sodium hypochlorite (SH) as a disinfectant. However, SH reacts with humic substances present in surface water, forming disinfection by-products (DBPs) regulated by the U.S. EPA. This study evaluated whether chitosan, a biopolymer with known bactericidal properties, could reduce chlorine demand and improve disinfection. Chitosan, with a 75% degree of deacetylation and a molecular weight of 460 kDa, was solubilized in four acids (acetic, citric, hydrochloric, and L-ascorbic) and tested under two turbidity ranges (236.0 and 2556 NTU). Chlorine demand curves were generated, and coliform presence–absence (P–A) tests were performed to assess bactericidal effects. Results showed that chitosan-treated samples achieved disinfection at the breakpoint with lower SH doses. For water with a turbidity of 236.0 NTU, all chitosan-acid solutions reached the breakpoint at 3.60 mg/L of SH. At 2556 NTU, three solutions required 4.20 mg/L SH, while hydrochloric acid–chitosan required only 3.60 mg/L. All chitosan-treated samples tested negative for coliforms, while controls treated with SH alone tested positive. These findings demonstrate that chitosan enhances bacterial removal and reduces chlorine demand, potentially lowering DBP formation in water treatment.

## 1. Introduction

Drinking water treatment in the United States is regulated by the Environmental Protection Agency (EPA), and drinking water must comply with established quality standards. A critical aspect of water purification is the elimination of pathogenic microorganisms that can cause infections and disease [1]. The Miradero Water Treatment Plant (MWTP) in Mayagüez, Puerto Rico, treats drinking water using sodium hypochlorite (SH) as a disinfectant. Chlorine, in its various forms, is the most widely used disinfectant due to its high reactivity and its ability to maintain a residual concentration throughout the water distribution system. While other disinfection methods can remove pathogens, they do not provide residual protection [2]. When chlorine is added to water, it undergoes several chemical reactions. Initially, it reacts with organic matter, resulting in no measurable residual chlorine and, thus, no disinfection. This stage is referred to as pre-chlorination, which plays a critical role in coagulation and flocculation, as surface water often contains high levels of natural organic matter. Pre-chlorine is measured hourly as a regulatory parameter. In the second stage, chlorine reacts with nitrogenous compounds, producing chloramines and some residual chlorine. The final disinfection phase begins after filtration, where an additional chlorine dose is applied to eliminate complex organic compounds, reduce odors, and generate the required residual chlorine concentration. This residual chlorine must persist for a defined contact time to ensure microbial inactivation [2]. Residual chlorine is essential in drinking water systems because it ensures ongoing microbial control in the distribution network. Treatment plants monitor residual chlorine in both treated and distributed water to comply with EPA standards.

Most drinking water treatment plants (WTPs) source water from rivers or lakes, which contain natural organic matter (NOM), a product of biological and chemical processes such as vegetation decay. NOM includes organic precursors like humic and fulvic acids, which can react with chlorine to form disinfection by-products (DBPs) [3,4]. These DBPs are regulated by the EPA due to their potential carcinogenicity [5,6]. Balancing effective disinfection while minimizing DBPs is a major challenge for water utilities [1].

Humic substances, formed through soil degradation, represent a significant fraction of NOM and contribute to 60–90% of the dissolved organic carbon in surface water [7]. Chlorine, being a strong oxidizing agent, effectively inactivates pathogens but also reacts with these compounds to form DBPs [8].

Biopolymers with bactericidal properties, such as chitosan, offer a promising alternative for reducing chlorine demand while maintaining disinfection efficiency. Chitin, a natural polymer derived primarily from crustaceans, can be deacetylated using concentrated NaOH or KOH solutions at high temperatures to produce chitosan [9]. Chitosan is the second most abundant natural polymer after cellulose [10]. It is a high molecular weight, cationic polysaccharide and is insoluble in water but soluble in acidic solutions due to the protonation of its amino groups. Its structure consists of β-(1-4)-D-glucosamine monomers [11]. Chitosan exhibits antibacterial activity due to its positive charge, which facilitates binding to negatively charged bacterial cell walls. A higher degree of deacetylation enhances this activity [12]. Solubility in the acid medium is crucial for achieving effective coagulation, flocculation, and microbial inactivation. The choice of acid influences chitosan’s performance, with low-carbon-number acids such as acetic and hydrochloric acid being most effective [10,13].

Previous research has demonstrated chitosan’s antimicrobial activity against bacteria, fungi, and yeast [14]. Ming Kong (2010) identified four factors that influence this activity: microbial characteristics, the charge density of chitosan, physical state (solid or liquid), and environmental conditions [14]. Nan Liu (2006) reported that chitosan in acetic acid showed bactericidal effects against E. coli at concentrations above 200 ppm, with greater effects observed for lower molecular weights [15]. Eaton (2008) similarly found that antimicrobial activity depends on molecular weight due to the varying mechanisms of action [16].

Chitosan interacts differently with Gram-positive and Gram-negative bacteria. In Gram-negative bacteria, it binds to anionic structures such as lipopolysaccharides, while in Gram-positive bacteria, it binds to peptidoglycans and teichoic acids [17]. Studies by Guarnieri and others concluded that the molecular weight and degree of deacetylation are the key determinants of antimicrobial effectiveness [15]. *Escherichia coli* (*E. coli*), a Gram-negative bacterium from the *Enterobacteriaceae* family, is widely used as an indicator of fecal contamination in water systems and is a common cause of infections [18].

Pontius (2016) demonstrated that chitosan-based coagulants can effectively reduce turbidity and bacterial contamination in drinking water. Chitosan was found to be a viable ecological alternative to traditional chemical coagulants like aluminum sulfate and ferric chloride [11].

The objective of this study was to evaluate the bactericidal effect of chitosan on the chlorine demand curve at the MWTP. Specifically, this study aimed to determine whether chitosan can enhance bacterial elimination during treatment and reduce the required sodium hypochlorite dose to achieve the desired residual chlorine levels. To this end, the chlorine demand was evaluated in the presence of chitosan dissolved in various acid media. In addition, the antimicrobial activity of chitosan was assessed using coliform presence–absence (P–A) tests.

## 2. Materials and Methods

### 2.1. Materials

Commercial chitosan powder (C3646), obtained from shrimp shells, was acquired from Sigma-Aldrich (St. Louis, MO, USA), with the percent of deacetylation degree (DD) ≥ 75% and an unknown molecular weight. L-ascorbic acid 99% (255564), sodium hydroxide (1310-73-2), and acetic acid 99.7% (64-19-7) were acquired from Sigma-Aldrich (USA). Citric acid (77-92-9) and hydrochloric acid 37% (7732-01-0) were acquired from Acros Organics (Burlington, MA, USA). The viscometer used was a Cannon-Ubbelohde [19] from the Chemistry Department, University of Puerto Rico at Mayagüez. Presence–absence (P–A) coliform tests were used to establish the presence of bacteria (coliforms). To analyze residual chlorine, a Hach Pocket Colorimeter [20] was used and a HQ40d Hach Meter [21] was used to analyze the pH and temperature. The jar test, with 1 L jars used, was performed by MCR Technologies [22].

### 2.2. Determining the Percent of Deacetylation Degree (DD)

To determine the DD, the potentiometric titration curve method was used Chitosan powder was dissolved in strong acid, such as 0.1 M Hydrochloric acid, and used along with 0.1 M NaOH as titrant. When solid chitosan is added to a solution of a strong acid, the NH_2_ amino groups of chitosan are ionized with the hydronium groups of the H_3_O^+^ acid, and the following reaction occurs:

R-NH_2_ + H_3_O^+^ => R-NH_3_^+^ (1)

By titrating the reaction with the strong base NaOH, which is dissociated into its Na^+^ and OH^−^ ions, these ions react first with the excess acid, that is, the hydronium ions, and then the protonation process of the NH_2_ amino groups of the chitosan occurs, shown in the following equation:

H_3_O^+^ + OH^−^ => H_2_O (2)

R-NH_3_^+^ + OH^−^ => R-NH_2_ (3)

The procedure to determine the DD involves adding solid chitosan in an acid solution with a known concentration, i.e., 0.4 g of chitosan in 30 mL of 0.1 M HCl. The solution was diluted up to 100 mL with distilled water. It was placed on a magnetic stirrer for 60 min, applying heat to ensure good dilution, and stored for 24 h. The chitosan was titrated with 0.1 M NaOH and the pH values were recorded at every 0.5 mL volume interval. The potentiometric curve obtained represents two inflection points because the solution had an excess of acid. The first point inflection corresponded to the neutralization point of the strong titrant base with the excess of strong acid in the solution, which had not yet protonated the amino groups. Then, the second inflection showed the neutralization of the already protonated chitosan [23,24,25]. To determine the DD% of chitosan, the following equations were used [26]:

DD% = [1 − 161Q]/[1 + 42Q](4)

Q = [C_NaOH_ (∆V)]/m (5)

where C_NaOH_ is the concentration of the titrant NaOH base, in mole per liter (mol/L). ∆V = V_2_ − V_1_, where V_2_ is the volume of the second inflection and V_1_ is the volume of the first inflection, in liters (L). The variable m is the weight of solid chitosan added to the acid solution, in grams (g). The molecular weight, in grams per mole (g/mol), of the monomer 2-amino-2-deoxy-β-D-glucosamine is 161, and 42 is the difference of molecular weights (203 g/mol, the molecular weight of 2-acetylamine-2deoxy-β-D-(+)-glucopyranose, minus 161 g/mol, the molecular weight of the monomer 2-amino-2-deoxy-β-D-glucosamine) [24,26].

### 2.3. Determining the Molecular Weight (Mw)

Chitosan powder (100 mg) was added to 10 mL of 0.25 M acetic acid/0.25 M sodium acetate [27,28] on a magnetic stirrer for 30 min to ensure good dilution. The chitosan and acetic acid solvent solution was diluted up to 100 mL with distilled water and stored for 24 h.

**Figure 1:**
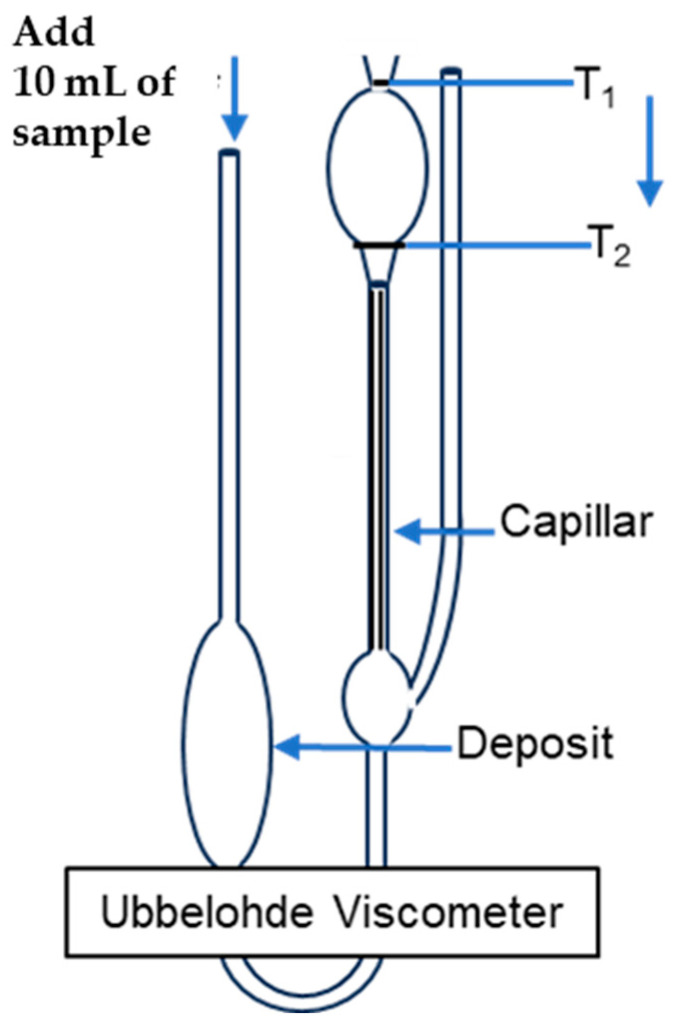
Ubbelohde viscometer, used to measure the time in which the solvent rose from mark T_1_ to mark T_2_.

The solvent (10 mL) was added in the Ubbelohde viscometer using a glass pipette. Using the pipette bulb, air was added to make the fluid rise to the T_1_ mark from the reservoir. With a digital chronometer (±0.01 s), the time in which the solvent rose from mark T_1_ to mark T_2_ was measured, according to Figure 1.

The same timing measurement process was performed three consecutive times with the sample and the solvent alone. The Higgins method was applied to the average sample and solvent times to determine the intrinsic viscosity. Equation (6) and Mark–Houwink constants, reported in the literature based on studies carried out with different types of solvents [28], were used.

Molecular weight is related to intrinsic viscosity, which is the ability of a polymer molecule to increase the viscosity of a solvent when it does not have intermolecular interactions. To determine the intrinsic viscosity (η), the Mark–Houwink equation was used [27]:

(η) = KM*^α^* (6)

where K and *α*, are Mark–Houwink constants and M is the molecular weight in grams per mole. Mark–Houwink constants have been reported in the literature based on studies carried out with different types of solvents, pH and molarity of the solution, and the DD range of chitosan [28].

### 2.4. Chitosan Stock Solutions—Preparation Procedure

Chitosan powder (100 mg) was added to 10 mL of four different acid solutions in 100 mL volumetric flasks. The acids used were acetic acid (0.1 M), citric acid (0.02 M), hydrochloric acid (0.1 M), and L-ascorbic acid (0.1 M). Each solution was placed on a magnetic stirrer for 30 min to ensure good dilution. In the citric, hydrochloric, and L-ascorbic acid solutions, heat was applied. Each solution was filled up to 100 mL with distilled water and stored for 24 h before usage. These were the stock solutions with a 1000 mg/L concentration of chitosan in each volumetric flask.

### 2.5. Analyzing the Levels of Chlorine Doses Required for Disinfection Before and After Adding Chitosan During the MWTP Process

#### 2.5.1. Collection and Preparation of Samples

Ten raw water samples (1000 mL each) were collected from the official MWTP sampling points and placed into 1 L jars. The following were added to each jar:

- A consistent dose of chitosan acid solution (mg/L);
- Varying doses of sodium hypochlorite (SH) (mg/L).

#### 2.5.2. Jar Test Analysis

The jar test was conducted following a structured mixing process:

- Rapid mixing: 1 min at 100 revolutions per minute (RPM).
- Slow mixing: 32 min at 30 RPM.
- Sedimentation phase: 178 min at 0 RPM.

This process replicated the actual treatment method used at MWTP.

#### 2.5.3. Sample Analysis

Samples were analyzed in triplicate at 178 min using the following methods:

- Residual chlorine measurement using the 4500-Cl(E) method. This is an EPA approved colorimeter and a standardized operational method [29].
- Temperature and pH measurement using an HQ40d Hach Meter [23].
- Presence–absence (P–A) coliform test, EPA Method 9221(B) [30], was used to determine the coliform presence. For the control samples (sodium hypochlorite only), the jar analyzed corresponded to the breakpoint stage, and sample volumes of 0.1 mL, 1.0 mL, and 10 mL were added to the lactose broth. For the chitosan-treated samples, three stages were analyzed: the jar before the breakpoint (using 0.1 mL of sample), the jar at the breakpoint (using 0.1 mL, 1.0 mL, and 10 mL), and the jar after the breakpoint (using 10 mL of sample), all added to the lactose broth. Water samples were collected in sterile containers containing sodium thiosulfate to neutralize any residual chlorine. Each sample was inoculated into a P–A broth medium containing lactose (as a fermentable sugar), nutrient salts, bromocresol purple as a pH indicator, and a selective agent to inhibit non-coliform bacteria. The inoculated tubes were incubated at 35 ± 0.5 °C for 24–48 h. A positive result was indicated by a color change from purple (neutral pH) to yellow, due to acid production from lactose fermentation by coliform bacteria.

Result Indicators:
**Color****Interpretation**PurpleNegative—No coliforms detectedYellowPositive Coliforms present (acid production)

- Additional microbiological verification using phenol red indicator in a lactose broth (control samples only): As a complementary analysis to the standard 9221 B Presence–Absence (P–A) test, an alternative lactose broth method using phenol red as a pH indicator was employed exclusively for control samples dosed with sodium hypochlorite (SH). This procedure served to confirm coliform presence in samples where SH was used alone, without chitosan. Six breakpoint jars were analyzed using this method, corresponding to the raw water samples with turbidities of 35.5, 236.0, and 2556 NTU (two jars per turbidity level). Each sample was inoculated into lactose broth containing phenol red and incubated at 35 ± 0.5 °C for 24–48 h. A positive result was indicated by a color change from red to yellow, due to acid production from lactose fermentation by coliform bacteria, similar to the interpretation criteria used in the 9221 Method. This colorimetric shift served as visual confirmation of bacterial activity in SH-treated samples. This complementary test was included to validate the results obtained from the standard method and to reinforce the observation of coliform presence in the SH-only control jars.

#### 2.5.4. Repetition with Variation

The entire process was repeated, with the use of different chitosan acid solutions. SH was added in different doses, keeping the dose of the chitosan solution constant, with 2.00 mg/L for the first turbidity range and 2.50 mg/L for the second range, as shown in Table 1.

## 3. Results and Discussions

### 3.1. Determining the Deacetylation Degree (DD) of Chitosan

The degree of deacetylation was determined using the potentiometric titration method by measuring the amino group content in the chitosan sample. A chitosan solution was prepared by dissolving 400 mg of chitosan powder in 30 mL of 0.1 M hydrochloric acid (HCl). The solution was then titrated with NaOH 0.1 M, recording the pH at 0.5 mL volume intervals while noting the corresponding NaOH volume added.

**Figure 2:**
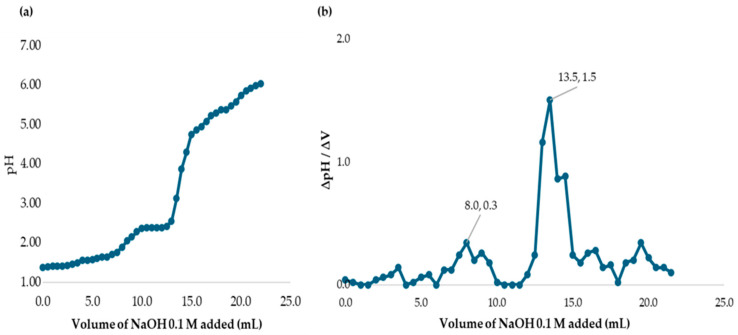
(**a**) Potentiometric titration of chitosan in HCl solution with NaOH. (**b**) Back titration curve of chitosan in HCl with NaOH.

The difference between the two inflection points in Figure 2 represents the amount of acid necessary to protonate the amine group in chitosan. Using Equations (4) and (5), the percentage of DD was calculated. The variable C_NaOH_, the concentration of the titrant NaOH base, was 0.1 mol/L. For the term ∆V = V2 − V1, V2 was 13.5 mL (0.0135 L) and V1 was 8.0 mL (0.008 L). The m was the 0.400 g of solid chitosan added to the acid solution. As such, ∆V = (13.5 mL − 8.0 mL)/1000 = 0.0055 L. Substituting all values into equations, the DD% value obtained was 75% which is the value reported by the manufacturer.

### 3.2. Determining the Molecular Weight (M)

The chitosan in solution with 0.25 M acetic acid/0.2 5 M sodium acetate (10 mL) was added in the Ubbelohde viscometer using a glass pipette at a temperature of 25 °C. Using the pipette bulb, air was added to make the fluid rise to the T1 mark from the reservoir.

Using a digital chronometer, the time it took for the solvent to move from mark T1 to mark T2 was measured, as shown in Figure 1. This timing measurement was repeated three consecutive times for both the sample and the solvent alone. Average flow times for both the chitosan solution and the solvent, used to apply the Higgins method, are presented in Table 2. These values were used to determine the intrinsic viscosity of chitosan. The example below illustrates the calculation process for a chitosan concentration of 1.000 × 10^−3^ g/mL, including the determination of relative viscosity, specific viscosity, reduced viscosity, and inherent viscosity:

η relative viscosity = (t solvent/t solution)(7)

η relative viscosity = 2.610/1.700 = 1.535

η specific viscosity = [(t solution − t solvent)/t solvent](8)

η specific viscosity = [(2.610 − 1.700)/1.700] = 0.535

η reduced viscosity = η specific viscosity/C(9)

η reduced viscosity = 0.535/0.001 g/mL = 535.2 mL/g

η inherent viscosity = ln (η relative viscosity)/C(10)

η inherent viscosity = ln (1.535)/0.001 = 428.7 mL/g

where:

t_solution = average flow time of chitosan solution.

t_solvent = average flow time of solvent.

C = chitosan concentration in g/mL.

**Figure 3:**
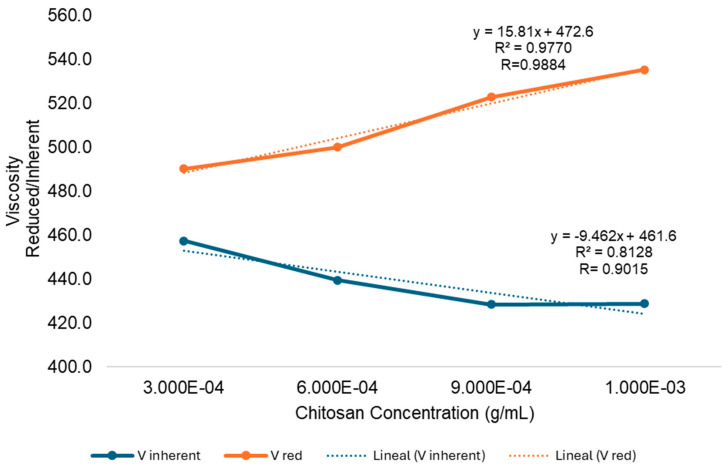
Reduced viscosity/Inherent viscosity vs. chitosan concentration in acetic acid solution.

The molecular weight is related to the intrinsic viscosity, which is the ability of a polymer molecule to increase the viscosity of a solvent when it does not have intermolecular interactions. To determine the intrinsic viscosity (η), the Mark–Houwink equation was used [20]. Figure 3 shows the average intercept of the reduced and inherent curves where η = average intercept = (461.6 + 472.6)/2 = 467.1.

Mark–Houwink constants have been reported in the literature based on studies carried out with different types of solvents [21]. Using the 0.25 M acetic acid/0.25 M sodium acetate as the solvent, the value of K and *α* was 15.7 × 10^−3^ and 0.79, respectively, and M was 460 kilodaltons (KDa). From Equation (6), as follows:

(η) = KM^*α*^

M = [(η)/K]^1/*α*^

M = [467.1/15.7 × 10^−3^]^1/0.79^

M = 460 KDa

### 3.3. Chlorine Demand of MWTP at Different Raw Water Turbidities Without Chitosan Added

The chlorine demand was calculated as the difference between the sodium hypochlorite (SH) dose and the residual chlorine concentration measured at 178 min using Equation (11):

Chlorine Demand (mg/L) = SH Dose (mg/L) − Residual Chlorine (mg/L)(11)

As shown in Table 3, the chlorine demand increased with the turbidity level and SH dose. For each turbidity condition, the table presents the corresponding residual chlorine and calculated chlorine demand, allowing for comparison across treatment conditions.

**Figure 4:**
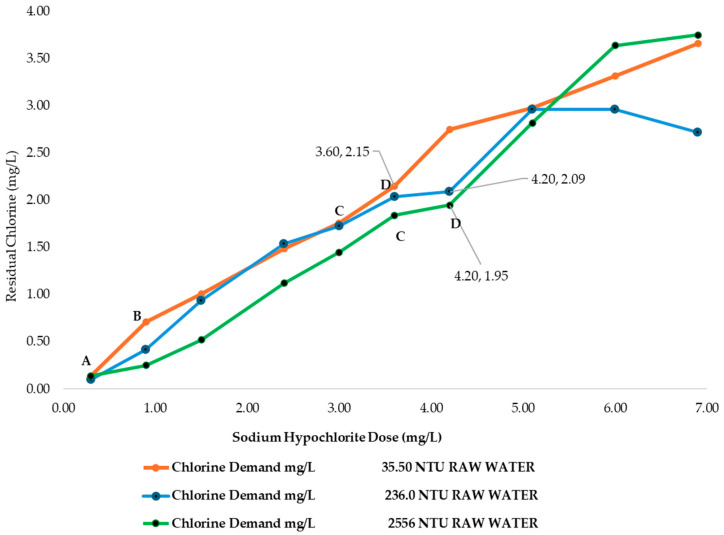
Chlorine demand curves at MWTP for different turbidities of raw water without chitosan.

Figure 4 illustrates the different stages of chlorination, labeled as stages AB, BC, and CD, using the chlorine demand curves generated at the MWTP under varying raw water turbidities. In Stage AB, point A represents the initial addition of sodium hypochlorite, where it began reacting with the organic matter present in the raw water. From point A to B, the residual chlorine remained near zero in all the curves shown in Figure 4, regardless of turbidity levels, indicating that reducing the compounds in the water was still consuming the chlorine before any measurable residual was established.

In Stage BC, there was a sharp increase in chlorine demand across all the curves shown in Figure 4. This occurred because nitrogenous compounds, a complex group of organic compounds, combined with chlorine, leading to a measurable residual chlorine level in the colorimeter. This stage represents the sum of residual chlorine from sodium hypochlorite and nitrogenous compounds. The increase in CD concentrations became more pronounced as the raw water turbidity decreases. At this stage, the difference between the samples became evident, as the lower organic composition resulted in less active chlorine available for disinfection. At the MWTP, no noticeable ammonia odor was detected at this point. The residual chlorine at this stage was in a combined form, as illustrated by the following reactions:

NH_3_ + HOCl = NH_2_Cl + H_2_O (12)

NH_2_Cl + HOCl = NHCl_2_ + H_2_O (13)

NHCl_2_ + HOCl = NCl_3_ + H_2_O (14)

Stage CD in Figure 4 represents the post-chlorination. At the MWTP, post chlorination occurs at the entrance of the Clearwell units. During this stage, the added chlorine reacts again with organic compounds, further improving the water quality by breaking down chloramine and chlorinated organic compounds. As a result, residual chlorine levels decrease due to its consumption in the process. However, at this point, the required pathogen disinfection to make the water potable has not yet been fully achieved. Figure 4 shows a significant difference between the low turbidity curve and the others, indicating that higher load of chlorinated compounds increased the chlorine demand at this disinfection stage.

Point D in Figure 4 represents the breakpoint, where the amount of chlorine added to the water results in a sustained residual level. At this stage, nitrogenous compounds have been completed destroyed. The residual chlorine can now be maintained throughout the distribution system, ensuring continued disinfection against contaminants that may enter the pipes and storage tanks outside the treatment plant. The breakpoint was more clearly observed in the curves corresponding to the raw water turbidities of 236.0 and 2556 NTU. In contrast, for the 35.50 NTU curve, the breakpoint appeared at a lower sodium hypochlorite dose, as the pathogen load was significantly smaller. At the MWTP, the sedimentation process is completed at 178 min, after which the water moves to the filtration units. At this point, only the combined residual chlorine was detected by the colorimeter. It is important to note that this data serve as a reference, as the chlorine demand curve were generated without considering other chemicals typically used in the drinking water treatment process. However, these curves effectively illustrate the behavior of sodium hypochlorite in the MWTP raw water.

**Figure 5:**
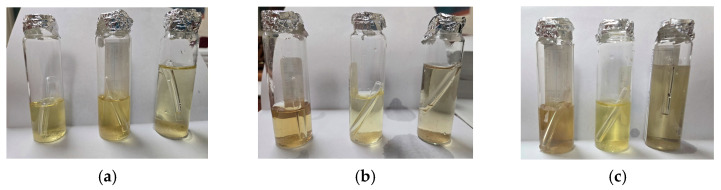
Positive results (yellow coloration) indicate acid production from lactose fermentation by coliform bacteria. (**a**) Coliform test results for sodium hypochlorite (SH) control samples at 35.5 NTU. (**b**) Coliform test results for SH control samples at 236.0 NTU. (**c**) Coliform verification results for samples at 2556 NTU.

Coliform testing results indicated that the samples analyzed tested positive for pathogenic microorganisms (breakpoint jar at 35.5, 236.0, and 2556 NTU), suggesting that additional treatment steps are necessary to achieve complete disinfection. To complement the microbiological assessment, an additional verification using lactose broth containing phenol red as a pH indicator was carried out exclusively on the control samples. Figure 5a–c shows the coliform results of the breakpoint jars at 35.5, 236.0, and 2556 NTU.

### 3.4. Chlorine Demand at MWTP for Different Raw Water Turbidities with the Addition of Chitosan

Acids with a low carbon number have been shown to be better solvents for chitosan [13]. This study evaluated the behavior of sodium hypochlorite (SH) as a disinfectant, in different acidic media and its bactericidal effects by coliform test analysis.

Four acids—acetic, hydrochloric, L-ascorbic, and citric—were used as solvents for chitosan, each with different strengths and pKa values. The underlying hypothesis was that the bactericidal effect of chitosan would remain strong under various conditions. The pKa values of chitosan in these acids were determined with potentiometric titration.

#### 3.4.1. Chitosan in Acetic Acid

Acetic acid is a monoprotic acid, and its pKa was determined in this study. Chitosan is considered a weak base insoluble in water but soluble in acids. It has a pKa of approximately 6.30 in acidic media with pH below 6.00.

**Figure 6:**
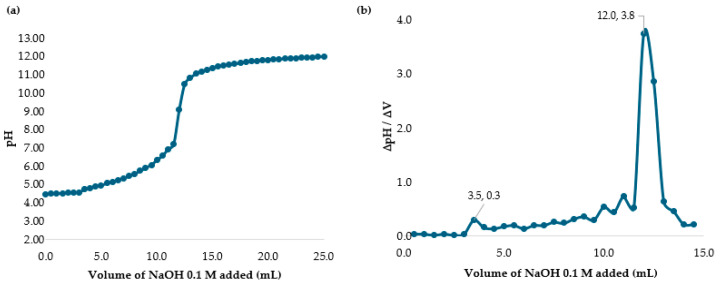
(**a**) Potentiometric titration of chitosan in acetic acid solution with NaOH. (**b**) Back titration curve of chitosan in acetic acid with NaOH.

Figure 6 shows the values of Volume 1 and 2, and a pKa of 5.54 for chitosan in acetic acid. This result indicated that the protonation of the amino groups of the polymer chain of chitosan can promote electrostatic interactions between the polymer and the cell walls of microorganisms. As a result, micro-floc formation was achieved, along with bactericidal effects.

Table 4 presents the sodium hypochlorite and chitosan dosages, along with the residual chlorine concentrations measured during the flocculation and sedimentation stages of the jar test at medium-high turbidity (236.0 NTU). All jars used the same chitosan dose (2.00 mg/L), while SH dose increased gradually, and the chlorine demand (CD) was calculated as the difference between the sodium hypochlorite (SH) dose and the residual chlorine (RC) concentration measured at 178 min.

**Figure 7:**
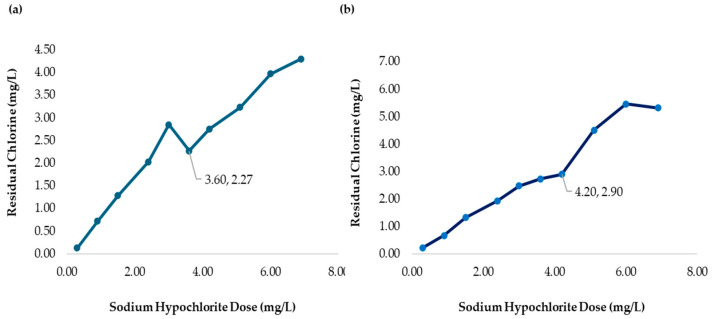
(**a**) Chlorine demand from the MWTP jar test analysis with the addition of chitosan in acetic acid (2.00 mg/L). The MWTP raw water turbidity was 236.0 NTU. (**b**) Chlorine demand from the MWTP jar test analysis with the addition of chitosan in acetic acid (2.50 mg/L). The MWTP raw water turbidity was 2556 NTU. Figures (**a**,**b**) show the jar test results from a 178 min process.

**Figure 8:**
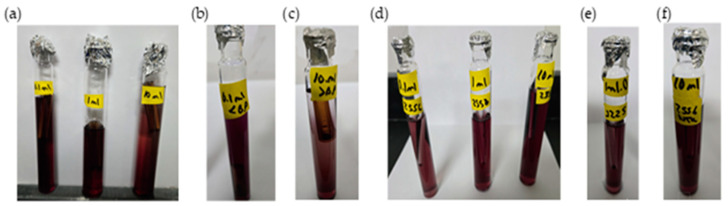
Coliform test results of chitosan (acetic acid)-treated samples using lactose broth. (**a**) Breakpoint jar results for samples with 236.0 NTU raw water (RW) using 0.1 mL, 1.0 mL, and 10 mL sample volumes. (**b**) Pre-breakpoint jar results (0.1 mL sample from 236.0 NTU RW). (**c**) Post-breakpoint jar results (10 mL sample from 236.0 NTU RW). (**d**) Breakpoint jar results for 2556 NTU RW using 0.1 mL, 1.0 mL, and 10 mL sample volumes. (**e**) Pre-breakpoint jar results (0.1 mL sample from 2556 NTU RW). (**f**) Post-breakpoint jar results (10 mL sample from 2556 NTU RW).

The chlorine demand curve shown in Figure 4 (236.0 NTU, without chitosan) exhibited a breakpoint at an SH dose of 4.20 mg/L, achieving a CD concentration of 2.09 mg/L and 1.33 mg/L of RC. In contrast, when chitosan in acetic acid (2.00 mg/L) was used, the SH dose required to reach the breakpoint was reduced to 3.60 mg/L, with a CD of 2.27 mg/L chlorine consumed during treatment, and the RC at this point was 1.33 mg/L, as shown in Figure 7a. This indicated that chitosan in acetic acid was effective in the oxidation of reducing substances present in the water, thereby lowering the SH dose needed to achieve complete disinfection. The coliform test was conducted on jars 1, 6, and 8 with all samples yielding negative results, as represented in Figure 8a–c.

Table 5 presents the sodium hypochlorite and chitosan dosages, along with the residual chlorine concentrations measured during the flocculation and sedimentation stages of the jar test at high turbidity (2556 NTU). All jars used the same chitosan dose (2.50 mg/L), while SH doses increased gradually, and the chlorine demand was calculated as the difference between the sodium hypochlorite (SH) dose and the residual chlorine (RC) concentration measured at 178 min. The chlorine demand curve shown in Figure 4 (2556 NTU, without the addition of chitosan) exhibited a breakpoint at an SH dose of 4.20 mg/L, resulting in a CD concentration of 1.95 mg/L with 2.25 mg/L of RC. Similarly, Figure 7b shows that when chitosan in acetic acid (2.50 mg/L) was added, the SH dose required to reach the breakpoint remained at 4.20 mg/L; however, a CD of 2.90 mg/L and 1.30 mg/L of RC was reached. This suggested that there is potential to optimize both the SH dose and chitosan concentration to reach the breakpoint at a lower dose. The coliform test was conducted on jars 1, 7, and 10, with all samples yielding negative results as shown in Figure 8d–f.

#### 3.4.2. Chitosan in Citric Acid

**Figure 9:**
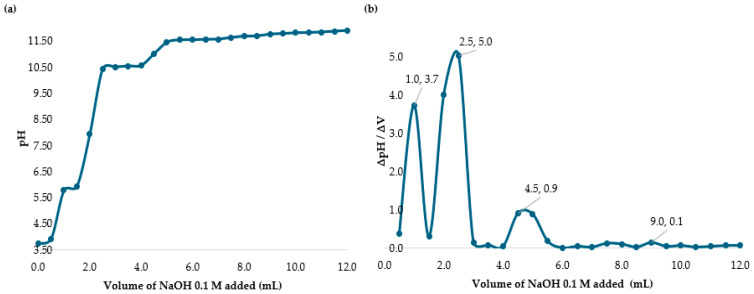
(**a**) Potentiometric titration of chitosan in citric acid with NaOH. (**b**) Back titration curve of chitosan in citric acid with NaOH.

Citric Acid is a polyprotic acid, and its pKa1, pKa2, and pKa3 were determined. Figure 9a shows the results with pKa1 = 6.75, pKa2 = 10.50, and pKa3 = 11.50, while Figure 9b presents the NaOH volumes used for these calculations.

Since citric acid has multiple ionizable protons, it undergoes protonation in phases, releasing protons stepwise. As the acid loses a proton, the resulting species becomes more negatively charged, making it progressively harder to lose additional protons due to electrostatic repulsion. Despite the pKa values being greater than six, the formation of microflocs was achieved, and bactericidal effects were observed, as shown in Figure 8.

Table 6 presents the sodium hypochlorite and chitosan (citric acid) dosages, along with the residual chlorine concentrations measured during the flocculation and sedimentation stages of the jar test at medium-high turbidity (236.0 NTU). All jars used the same chitosan dose (2.00 mg/L), while SH dose increased gradually, and the chlorine demand (CD) was calculated as the difference between the sodium hypochlorite (SH) dose and the residual chlorine (RC) concentration measured at 178 min.

**Figure 10:**
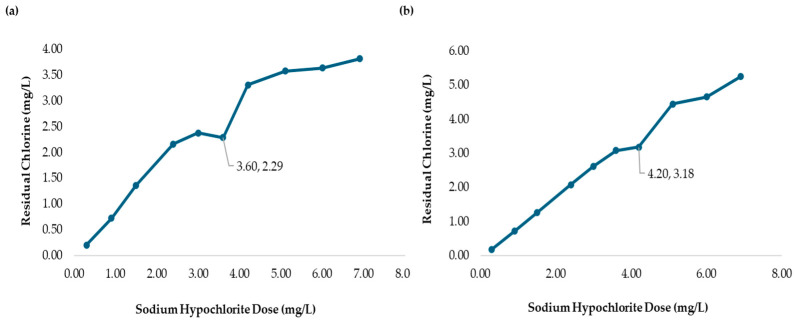
(**a**) Chlorine demand from the MWTP jar test analysis with the addition of chitosan in citric acid (2.00 mg/L). The MWTP raw water turbidity was 236.0 NTU. (**b**) Chlorine demand from the MWTP jar test analysis with the addition of chitosan in citric acid (2.50 mg/L). The MWTP raw water turbidity was 2556 NTU. Figures (**a**,**b**) show the jar test results from a 178 min process.

**Figure 11:**
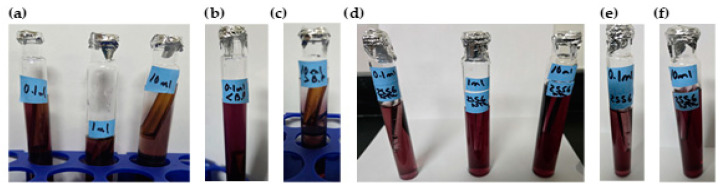
Coliform test results of chitosan (citric acid)-treated samples using lactose broth. (**a**) Breakpoint jar results for samples with 236.0 NTU raw water (RW) using 0.1 mL, 1.0 mL, and 10 mL sample volumes. (**b**) Pre-breakpoint jar results (0.1 mL sample from 236.0 NTU RW). (**c**) Post-breakpoint jar results (10 mL sample from 236.0 NTU RW). (**d**) Breakpoint jar results for 2556 NTU RW using 0.1 mL, 1.0 mL, and 10 mL sample volumes. (**e**) Pre-breakpoint jar results (0.1 mL sample from 2556 NTU RW). (**f**) Post-breakpoint jar results (10 mL sample from 2556 NTU RW).

Figure 10a presents the chlorine demand curve (236.0 NTU) for MWTP after adding chitosan in citric acid (2.00 mg/L). The breakpoint was reached at an SH dose of 3.60 mg/L, with CD of 2.29 mg/L. The demand curve for chitosan in citric acid achieved its breakpoint at a lower SH dose compared to Figure 4. The coliform test analysis for jars 1, 6, and 10 yielded negative results as shown in Figure 11a–c.

Table 7 presents the sodium hypochlorite and chitosan (citric acid) dosages, along with the residual chlorine concentrations measured during the flocculation and sedimentation stages of the jar test at high turbidity (2556 NTU). All jars used the same chitosan dose (2.50 mg/L), while SH dose increased gradually, and the chlorine demand was calculated as the difference between the sodium hypochlorite (SH) dose and the residual chlorine (RC) concentration measured at 178 min. Figure 10b shows the chlorine demand curve (2556 NTU) for the MWTP after adding chitosan in citric acid (2.50 mg/L). The breakpoint occurred at an SH dose of 4.20 mg/L, with a CD of 3.18 mg/L and 1.02 mg/L of RC. Chitosan in citric acid effectively oxidized the reducing substance, and the higher CD indicated potential for the further optimization of SH and chitosan concentration. This behavior was similar to the acetic acid curve. The coliform test for jars 1, 7, and 10 also yielded negative results as shown in Figure 11d–f.

#### 3.4.3. Chitosan in Hydrochloric Acid

**Figure 12:**
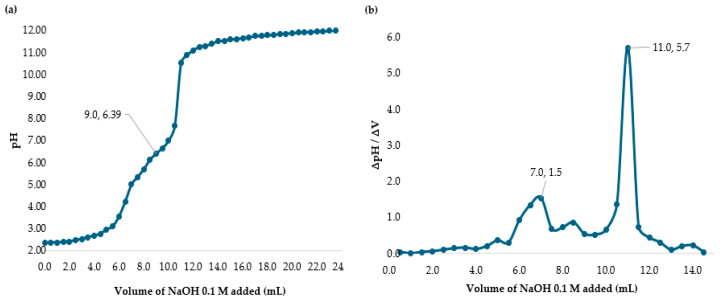
(**a**) Potentiometric titration of chitosan in hydrochloric acid with NaOH. (**b**) Back titration curve of chitosan in hydrochloric acid with NaOH.

Hydrochloric acid is a strong monoprotic acid. Figure 12a,b show Volumes 1 (7 mL) and 2 (11 mL), with a calculated pKa of 6.39, which is close to 6.00. While bactericidal effects were observed, they were lower compared to those in the acetic acid tests.

Table 8 presents the sodium hypochlorite and chitosan (hydrochloric acid) dosages, along with the residual chlorine concentrations measured during the flocculation and sedimentation stages of the jar test at medium-high turbidity (236.0 NTU). All jars used the same chitosan dose (2.00 mg/L), while SH dose increased gradually, and the chlorine demand (CD) was calculated as the difference between the sodium hypochlorite (SH) dose and the residual chlorine (RC) concentration measured at 178 min.

**Figure 13:**
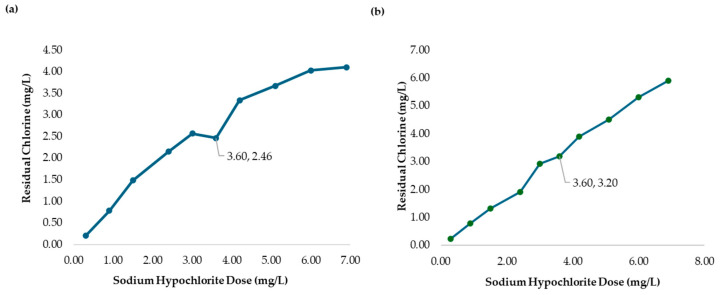
(**a**) Chlorine demand from the MWTP jar test analysis with the addition of chitosan in hydrochloric acid (2.00 mg/L). The MWTP raw water turbidity was 236.0 NTU. (**b**) Chlorine demand from the MWTP jar test analysis with the addition of chitosan in hydrochloric acid (2.50 mg/L). The MWTP raw water turbidity was 2556 NTU. Figures (**a**,**b**) show the jar test results from a 178 min process.

**Figure 14:**
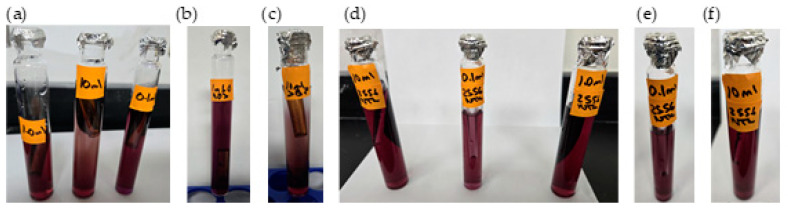
Coliform test results of chitosan (hydrochloric acid)-treated samples using lactose broth. (**a**) Breakpoint jar results for samples with 236.0 NTU raw water (RW) using 0.1 mL, 1.0 mL, and 10 mL sample volumes. (**b**) Pre-breakpoint jar results (0.1 mL sample from 236.0 NTU RW). (**c**) Post-breakpoint jar results (10 mL sample from 236.0 NTU RW). (**d**) Breakpoint jar results for 2556 NTU RW using 0.1 mL, 1.0 mL, and 10 mL sample volumes. (**e**) Pre-breakpoint jar results (0.1 mL sample from 2556 NTU RW). (**f**) Post-breakpoint jar results (10 mL sample from 2556 NTU RW).

Figure 13a presents the chlorine demand curve for the MWTP after adding chitosan in hydrochloric acid (2.00 mg/L). The breakpoint was reached at an SH dose of 3.60 mg/L, with 2.46 mg/L of CD and 1.14 mg/L of RC. The raw water turbidity was 236.0 NTU. The demand curve for chitosan in hydrochloric acid achieved its breakpoint at a lower dose than in Figure 4. The coliform test results for jars 1, 6, and 10 were negative as shown in Figure 14a–c.

Figure 13b illustrates a breakpoint at an SH dose of 3.60 mg/L, with a CD of 3.20 mg/L, when using hydrochloric acid (2.50 mg/L) with a raw water turbidity of 2556 NTU. Chitosan in hydrochloric acid effectively oxidized reducing substances, with the RC level indicating potential for optimizing the chitosan dosage to reach the breakpoint with higher SH dose. The coliform test results for jars 1, 6, and 10 were negative shown in Figure 14d–f.

Table 9 presents the sodium hypochlorite and chitosan (hydrochloric acid) dosages, along with the residual chlorine concentrations measured during the flocculation and sedimentation stages of the jar test at high turbidity (2556 NTU). All jars used the same chitosan dose (2.50 mg/L), while SH dose increased gradually, and the chlorine demand was calculated as the difference between the sodium hypochlorite (SH) dose and the residual chlorine (RC) concentration measured at 178 min.

#### 3.4.4. Chitosan in L-Ascorbic Acid

**Figure 15:**
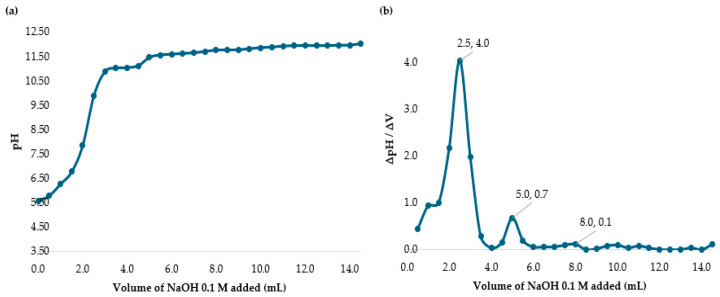
(**a**) Potentiometric titration of chitosan in L-ascorbic acid with NaOH. (**b**) Back titration curve of chitosan in L-ascorbic acid with NaOH.

L-ascorbic acid is a diprotic acid, with two determined pKa values. Figure 15a illustrates the volumes of NaOH added during titration, yielding two values: pKa1 = 11.01 and pKa2 = 11.75. Figure 15b represents these values with higher peaks, indicating the stepwise protonation process. Similar to citric acid, L-ascorbic acid releases its protons sequentially.

**Figure 16:**
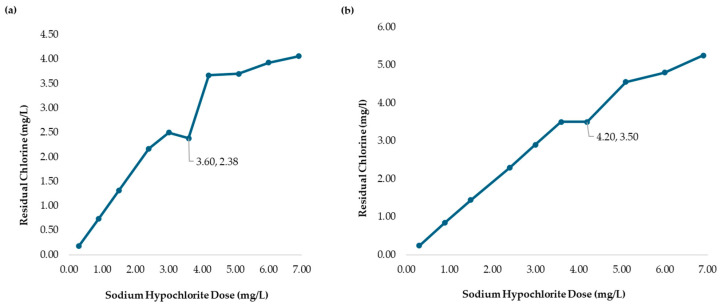
(**a**) Chlorine demand from the MWTP jar test analysis with the addition of chitosan in L-ascorbic acid (2.00 mg/L). The MWTP raw water turbidity was 236.0 NTU. (**b**) Chlorine demand from the MWTP jar test analysis with the addition of chitosan in L-ascorbic acid (2.50 mg/L). The MWTP raw water turbidity was 2556 NTU. Figures (**a**,**b**) show the jar test results from a 178 min process.

**Figure 17:**
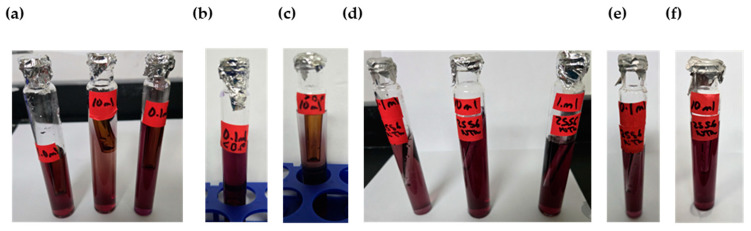
Coliform test results of chitosan (L-ascorbic acid)-treated samples using lactose broth. (**a**) Breakpoint jar results for samples with 236.0 NTU raw water (RW) using 0.1 mL, 1.0 mL, and 10 mL sample volumes. (**b**) Pre-breakpoint jar results (0.1 mL sample from 236.0 NTU RW). (**c**) Post-breakpoint jar results (10 mL sample from 236.0 NTU RW). (**d**) Breakpoint jar results for 2556 NTU RW using 0.1 mL, 1.0 mL, and 10 mL sample volumes. (**e**) Pre-breakpoint jar results (0.1 mL sample from 2556 NTU RW). (**f**) Post-breakpoint jar results (10 mL sample from 2556 NTU RW).

Although the pKa values exceed 6.00, microfloc formation was achieved, and bactericidal effects were observed, as shown in Figure 16a. The breakpoint was reached at an SH dose of 3.60 mg/L, with a CD of 2.38 mg/L, in raw water with a turbidity of 236.0 NTU. The demand curve for chitosan in L-ascorbic acid achieved its breakpoint at a lower SH dose than in Figure 4, and the coliform test results for jars 1, 6, and 10 were negative as shown in Figure 17a–c.

Table 10 shows the sodium hypochlorite and chitosan (L-ascorbic acid) dosages, along with the residual chlorine concentrations measured during the flocculation and sedimentation stages of the jar test at medium-high turbidity (236.0 NTU). All jars used the same chitosan dose (2.00 mg/L), while SH dose increased gradually, and the chlorine demand (CD) was calculated as the difference between the sodium hypochlorite (SH) dose and the residual chlorine (RC) concentration measured at 178 min.

Despite L-ascorbic acid having the highest pKa values among the tested acids, the required SH dose at breakpoint was comparable to the others. This suggests that its bactericidal properties contributed to achieving disinfection at a lower SH dose while maintaining an optimal residual chlorine level above 2.00 mg/L. Figure 16b presents a breakpoint at an SH dose of 4.20 mg/l with a CD of 3.50 mg/L when using chitosan in L-ascorbic acid (2.50 mg/L) with raw water at 2556 NTU. Table 11 presents the sodium hypochlorite and chitosan (hydrochloric acid) dosages, along with the residual chlorine concentrations measured during the flocculation and sedimentation stages of the jar test at high turbidity (2556 NTU). All jars used the same chitosan dose (2.50 mg/L), while SH dose increased gradually, and the chlorine demand was calculated as the difference between the sodium hypochlorite (SH) dose and the residual chlorine (RC) concentration measured at 178 min.

Chitosan in L-ascorbic acid effectively oxidized reducing substances, and the higher residual chlorine level suggested potential for optimizing the SH dose while maintaining effective disinfection. The coliform test results for jars 1, 7, and 10 were all negative shown in Figure 17d–f.

## 4. Conclusions

This study demonstrated that using chitosan in an acid solution enhances its bactericidal effect, aiding in bacterial elimination and allowing the disinfection point (breakpoint) to be reached with a lower dose of sodium hypochlorite (SH). It highlights how chitosan’s antimicrobial properties function in the raw water at the MWTP under varying turbidity levels. These findings reinforce the previously described mechanisms of action of chitosan against Gram-negative bacteria, such as *E. coli*, which were discussed earlier in the introduction. In this study, chitosan, with a 75% degree of deacetylation and medium-high molecular weight (460 kDa), proved effective in eliminating coliforms across all the acidic solvents tested, even at varying turbidity levels. Notably, all chitosan-treated samples tested negative for coliforms, while the chlorine-only control remained positive. This outcome underscores the significance of chitosan’s interaction with bacterial cell walls, particularly in Gram-negative species, and provides experimental support for its antimicrobial efficacy under surface water treatment conditions, including high turbidity. These results align with those reported by Guarnieri et al. [17] and Liu et al. [15], highlighting the critical role of molecular structure in determining the biopolymer’s effectiveness.

Chitosan powder, with a validated 75% degree of deacetylation (DD) and medium-high molecular weight (460 kDa), effectively eliminated coliforms and reduced the pathogenic microorganism load targeted by the disinfectant. These findings suggest that chitosan can help lower chlorine demand during treatment, thereby minimizing the risk of chlorine-induced disinfection byproduct (DBP) formation.

The results further demonstrated that the addition of chitosan reduced chlorine demand and allowed the disinfection breakpoint to be reached at a lower SH dose. This aligned with the findings of Pontius (2016), who reported that chitosan acts as a coagulant with antimicrobial properties, enhancing the removal of both organic matter and pathogens [11]. Additionally, Edzwald (1985) emphasized the importance of surrogate parameters in predicting trihalomethane (THM) precursors [4]; this study contributed by showing how chitosan can mitigate these risks by reducing the required chlorine dose.

Furthermore, the residual chlorine concentrations observed remained below the EPA’s regulatory limit of 4.0 mg/L (EPA, 1998), suggesting that chitosan may help optimize disinfection while minimizing the formation of harmful disinfection byproducts (DBPs). Compared to traditional chemical treatments, the use of chitosan also presents a more environmentally friendly alternative, consistent with the conclusions of Kalita et al. who emphasized the health benefits of reducing DBP exposure in drinking water treatment [6].

**Figure 18:**
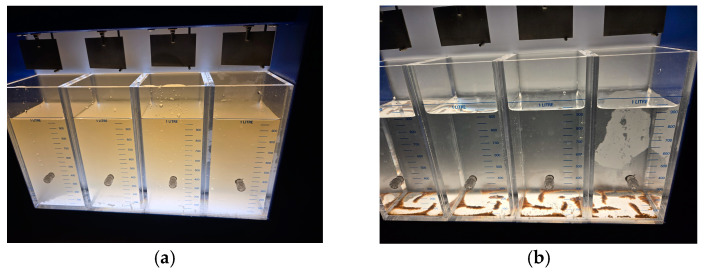
(**a**) Jar test using only sodium hypochlorite (SH). (**b**) Jar test after the addition of chitosan.

Across all the tested acids, chitosan exhibited similar behavior despite significant differences in solubility. Its bactericidal effect successfully reduced the chlorine demand curve and allowed disinfection to be achieved at the breakpoint with a lower SH dose. Coliform tests confirmed these bactericidal properties, as all chitosan-treated samples tested negative while those treated solely with chlorine tested positive for bacterial presence. Figure 18a shows the jar test for samples only with SH. Figure 18b shows the jar test with the samples after adding chitosan.

The four acidic chitosan solutions reached the breakpoint at an SH dose of 3.60 mg/L when the raw water turbidity was 236.0 NTU. At a turbidity of 2556 NTU, the SH dose required to reach the breakpoint was consistent across all curves (4.20 mg/L), except for chitosan dissolved in hydrochloric acid, which achieved the breakpoint at a lower SH dose (3.60 mg/L) while maintaining a residual chlorine concentration above 1.00 mg/L.

According to the EPA’s Stage 2 Disinfection Byproducts Rule, the maximum allowable residual chlorine (RC) concentration in drinking water is 4.0 mg/L to minimize the formation of potentially harmful disinfection byproducts (EPA, 1998) [1].

As part of disinfection byproduct (DBP) control strategies, water treatment plants are required to maintain a moderate residual chlorine level sufficient to ensure microbial safety throughout the distribution system, including its farthest points, where a minimum residual chlorine concentration of 0.2 mg/L must be sustained. In practice, achieving an RC between 1.0 and 2.0 mg/L has proven effective under the conditions present at MWTP. This suggests that chitosan is a viable alternative to help reduce SH dosing during treatment, thereby minimizing DBP formation both during the treatment process and within the distribution network. Moreover, the ability to achieve adequate disinfection while maintaining lower RC levels in the finished water supply supports chitosan’s potential to enhance compliance with regulatory limits and improve overall water safety.

Table 12 presents the comparative data of the sodium hypochlorite (SH) dosages and the corresponding residual chlorine (RC) concentrations obtained under different turbidity conditions in this study.

In this study, the residual chlorine concentrations (RC) remained below this regulatory threshold, with breakpoints achieved at doses as low as 3.60 mg/L. These findings suggest that incorporating chitosan into the treatment process may reduce chlorine demand while still ensuring effective disinfection. Table 3 shows residual chlorine (RC) at the breakpoint using chitosan in acidic solutions, compared to SH alone, at two raw water turbidity levels. Chitosan solubility varied among the acidic solutions, ranked as follows based on the pKa values:

Acetic > Hydrochloric > Citric > L-Ascorbic

**Figure 19:**
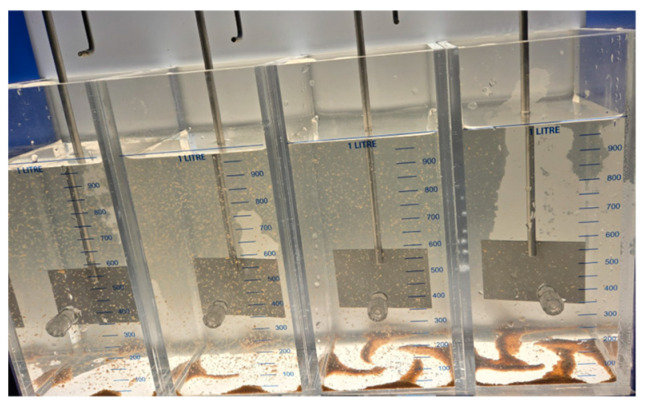
Jar test results during 30 min of flocculation at 30 RPM, using chitosan dissolved in (from **left** to **right**) L-ascorbic, citric acid, hydrochloric acid, and acetic acid, respectively.

Figure 19 displays the breakpoint jars for all the acid solvents, arranged in order of solubility, highlighting the differences observed in the flocculation process during the 30 min jar test (30 RPM). Chitosan dissolved in acetic acid formed flocs the fastest, showing visible settling even before the mixing stage ended. It was followed by hydrochloric acid, then citric acid, and finally L-ascorbic acid, which showed the slowest floc formation and settling.

Despite the differences in solubility, all acidic chitosan solutions effectively reached the chlorine demand breakpoint. Each acid solution demonstrated a reduced sodium hypochlorite (SH) dose requirement to achieve the breakpoint compared to the control curves without chitosan.

The average sodium hypochlorite consumption in water treatment plants with capacities similar to MWTP is estimated at 7000 lbs/day, which could result in monthly expenses exceeding $90,000. This study highlights the potential for cost savings by using biodegradable additives like chitosan to reduce chlorine demand, which could have a significant impact on operational costs. The results shown in Table 12 indicate that in the control sample (SH), 4.20 mg/L of SH was dosed to reach the breakpoint, with a residual chlorine (RC) concentration of 1.95 mg/L; however, this dose still showed the presence of coliforms. Therefore, using a bactericidal product like chitosan, while progressively optimizing the dosage, could lead to substantial savings for water treatment plants. Future studies could further evaluate these chitosan solutions in terms of turbidity reduction and total organic matter removal. By optimizing doses and comparing effectiveness, the role of chitosan’s bactericidal effects could be assessed independently of turbidity levels.

Chitosan is considered a cost-effective alternative or complement to traditional chemical disinfectants, especially when its dual functionality as a coagulant and antimicrobial agent is factored in. While the unit cost of chitosan is higher than that of sodium hypochlorite, its ability to reduce chlorine demand and minimize disinfection byproduct (DBP) formation can result in significant long-term savings in chemical consumption and compliance costs.

In this study, chitosan was effective at concentrations as low as 2.00–2.50 mg/L, depending on water turbidity levels. Based on standard dosing and bulk density, an estimated 2–3 kg of chitosan powder would be required per 1,000,000 L of treated water. The bulk market price of technical-grade chitosan ranges from $10 to $20 USD/kg, which translates to $20–60 USD per million liters. When compared with the potential chlorine cost savings (which may exceed $0.10 USD/m^3^, or $100 per million liters), the investment in chitosan is justified, especially in plants with high turbidity or high chlorine demand. Table 13 shows a comparative summary of the dosages and costs for chitosan and conventional water treatment chemicals [31]

Regarding safety, chitosan is non-toxic, biodegradable, and derived from natural sources, mainly crustacean shells. It has been studied extensively and is generally recognized as safe (GRAS) by the U.S. Food and Drug Administration (FDA) for use in food and medical applications. While it is not yet standardized as a disinfectant by the EPA, its use in water treatment has been validated in various peer-reviewed studies. To ensure safe application, quality standards such as heavy metal content, ash percentage, solubility, and microbiological purity should be evaluated prior to use. Additionally, the chitosan used in this study underwent degree of deacetylation and molecular weight characterization, both critical to ensuring its effectiveness and safety.

Future work may involve a life-cycle cost analysis comparing chitosan-enhanced treatment with traditional chlorination methods, as well as studies on residuals management and environmental impact.

## Figures and Tables

**Table 1:** Sodium hypochlorite (SH) dosage according to the turbidity range of raw water (RW).

		RW Turbidity Range	RW Turbidity Range
		30–250 (NTU)	250–2600 (NTU)
Jar	SH (mg/L)	Chitosan in Acidic Solution (mg/L)	Chitosan in Acidic Solution (mg/L)
1	0.30	2.00	2.50
2	0.90	2.00	2.50
3	1.50	2.00	2.50
4	2.40	2.00	2.50
5	3.00	2.00	2.50
6	3.60	2.00	2.50
7	4.20	2.00	2.50
8	5.10	2.00	2.50
9	6.00	2.00	2.50
10	6.90	2.00	2.50

(NTU: Nephelometric Turbidity Units, the standard measure of water clarity based on the scattering of light by suspended particles).

**Table 2:** Viscosity data.

	Acetic Acid (0.1 M)	Chitosan Concentrations (g/mL)			
	Solvent	1.000 × 10^−3^	9.000 × 10^−4^	6.000 × 10^−4^	3.000 × 10^−4^
t average (min)	1.700 ± 0.011	2.610 ± 0.013	2.500 ± 0.011	2.213 ± 0.017	1.950 ± 0.045
Relative viscosity		1.535	1.471	1.302	1.147
Specific viscosity		0.535	0.471	0.302	0.147
Reduced viscosity		535.3	522.8	500.0	490.2
Inherent viscosity		428.7	428.5	439.5	457.3

**Table 3:** Chlorine demand (CD) and residual chlorine (RC) following sodium hypochlorite dosing in jar test experiments at varying raw water turbidities.

			35.5 NTU		236.0 NTU		2556 NTU	
Jar	SH (mg/L)	SH (µL)	RC (mg/L)	CD (mg/L)	RC (mg/L)	CD (mg/L)	RC (mg/L)	CD (mg/L)
1	0.30	100	0.16	0.14	0.20	0.10	0.16	0.14
2	0.90	300	0.19	0.71	0.48	0.42	0.65	0.25
3	1.50	500	0.49	1.01	0.56	0.94	0.98	0.52
4	2.40	800	0.91	1.49	0.86	1.54	1.28	1.12
5	3.00	1000	1.24	1.76	1.27	1.73	1.55	1.45
6	3.60	1200	1.45	2.15	1.56	2.04	1.76	1.84
7	4.20	1400	1.45	2.75	2.11	2.09	2.25	1.95
8	5.10	1700	2.12	2.98	2.14	2.96	2.28	2.82
9	6.00	2000	2.68	3.32	3.04	2.96	2.36	3.64
10	6.90	2300	3.24	3.66	4.18	2.72	3.15	3.75

**Table 4:** Dosage and residual chlorine levels for sodium hypochlorite and chitosan (acetic acid) during jar test at 236.0 NTU Raw Water.

Jar	SH (mg/L)	SH (µL)	Chitosan (mg/L)	Chitosan (µL)	RC After Flocculation (30 min, 30 RPM)	RC After Sedimentation (178 min, 0 RPM)	CD (mg/L)
1	0.30	100	2.00	2000	0.20	0.17	0.13
2	0.90	300	2.00	2000	0.29	0.18	0.72
3	1.50	500	2.00	2000	0.38	0.22	1.28
4	2.40	800	2.00	2000	0.67	0.37	2.03
5	3.00	1000	2.00	2000	0.26	0.15	2.85
6	3.60	1200	2.00	2000	1.73	1.33	2.27
7	4.20	1400	2.00	2000	1.78	1.45	2.75
8	5.10	1700	2.00	2000	2.62	1.87	3.23
9	6.00	2000	2.00	2000	2.82	2.03	3.97
10	6.90	2300	2.00	2000	3.44	2.60	4.30

**Table 5:** Dosage and residual chlorine levels for sodium hypochlorite and chitosan (acetic acid) during jar test at 2556 NTU raw water.

Jar	SH (mg/L)	SH (µL)	Chitosan (mg/L)	Chitosan (µL)	RC After Flocculation (30 min, 30 RPM)	RC After Sedimentation (178 min, 0 RPM)	CD (mg/L)
1	0.30	100	2.50	2500	0.15	0.08	0.22
2	0.90	300	2.50	2500	0.29	0.23	0.67
3	1.50	500	2.50	2500	0.18	0.18	1.32
4	2.40	800	2.50	2500	0.84	0.47	1.93
5	3.00	1000	2.50	2500	1.01	0.52	2.48
6	3.60	1200	2.50	2500	1.08	0.87	2.73
7	4.20	1400	2.50	2500	1.97	1.30	2.90
8	5.10	1700	2.50	2500	1.29	0.59	4.51
9	6.00	2000	2.50	2500	1.34	0.54	5.46
10	6.90	2300	2.50	2500	3.48	1.58	5.32

**Table 6:** Dosage and residual chlorine levels for sodium hypochlorite and chitosan (citric acid) during jar test at 236.0 NTU Raw Water.

Jar	SH (mg/L)	SH (µL)	Chitosan (mg/L)	Chitosan (µL)	RC After Flocculation (30 min, 30 RPM)	RC After Sedimentation (178 min, 0 RPM)	CD (mg/L)
1	0.30	100	2.00	2000	0.23	0.10	0.20
2	0.90	300	2.00	2000	0.25	0.18	0.72
3	1.50	500	2.00	2000	0.64	0.14	1.36
4	2.40	800	2.00	2000	1.17	0.24	2.16
5	3.00	1000	2.00	2000	1.60	0.62	2.38
6	3.60	1200	2.00	2000	2.15	1.31	2.29
7	4.20	1400	2.00	2000	2.58	0.89	3.31
8	5.10	1700	2.00	2000	3.12	1.52	3.58
9	6.00	2000	2.00	2000	3.62	2.36	3.64
10	6.90	2300	2.00	2000	3.78	3.08	3.82

**Table 7:** Dosage and residual chlorine levels for sodium hypochlorite and chitosan (citric acid) during jar test at 2556 NTU raw water.

Jar	SH (mg/L)	SH (µL)	Chitosan (mg/L)	Chitosan (µL)	RC After Flocculation (30 min, 30 RPM)	RC After Sedimentation (178 min, 0 RPM)	CD (mg/L)
1	0.30	100	2.50	2500	0.09	0.12	0.18
2	0.90	300	2.50	2500	0.05	0.19	0.71
3	1.50	500	2.50	2500	0.11	0.24	1.26
4	2.40	800	2.50	2500	0.18	0.32	2.08
5	3.00	1000	2.50	2500	0.14	0.38	2.62
6	3.60	1200	2.50	2500	0.58	0.52	3.08
7	4.20	1400	2.50	2500	0.77	1.02	3.18
8	5.10	1700	2.50	2500	0.79	0.65	4.45
9	6.00	2000	2.50	2500	1.51	1.34	4.66
10	6.90	2300	2.50	2500	1.92	1.65	5.25

**Table 8:** Dosage and residual chlorine levels for sodium hypochlorite and chitosan (hydrochloric acid) during jar test at 236.0 NTU raw water.

Jar	SH (mg/L)	SH (µL)	Chitosan (mg/L)	Chitosan (µL)	RC After Flocculation (30 min, 30 RPM)	RC After Sedimentation (178 min, 0 RPM)	CD (mg/L)
1	0.30	100	2.00	2000	0.29	0.10	0.20
2	0.90	300	2.00	2000	0.31	0.12	0.78
3	1.50	500	2.00	2000	0.23	0.02	1.48
4	2.40	800	2.00	2000	0.55	0.25	2.15
5	3.00	1000	2.00	2000	0.81	0.43	2.57
6	3.60	1200	2.00	2000	1.45	1.14	2.46
7	4.20	1400	2.00	2000	1.82	0.86	3.34
8	5.10	1700	2.00	2000	2.94	1.43	3.67
9	6.00	2000	2.00	2000	3.44	1.97	4.03
10	6.90	2300	2.00	2000	4.80	2.80	4.10

**Table 9:** Dosage and residual chlorine levels for sodium hypochlorite and chitosan (hydrochloric acid) during jar test at 2556 NTU raw water.

Jar	SH (mg/L)	SH (µL)	Chitosan (mg/L)	Chitosan (µL)	RC After Flocculation (30 min, 30 RPM)	RC After Sedimentation (178 min, 0 RPM)	CD (mg/L)
1	0.30	100	2.50	2500	0.15	0.06	0.24
2	0.90	300	2.50	2500	0.26	0.11	0.79
3	1.50	500	2.50	2500	0.82	0.18	1.32
4	2.40	800	2.50	2500	1.82	0.48	1.92
5	3.00	1000	2.50	2500	2.35	0.07	2.93
6	3.60	1200	2.50	2500	3.22	0.40	3.20
7	4.20	1400	2.50	2500	3.36	0.31	3.89
8	5.10	1700	2.50	2500	3.98	0.59	4.51
9	6.00	2000	2.50	2500	4.01	0.69	5.31
10	6.90	2300	2.50	2500	4.15	0.99	5.91

**Table 10:** Dosage and residual chlorine levels for sodium hypochlorite and chitosan (L-ascorbic acid) during jar test at 236.0 NTU raw water.

Jar	SH (mg/L)	SH (µL)	Chitosan (mg/L)	Chitosan (µL)	RC After Flocculation (30 min, 30 RPM)	RC After Sedimentation (178 min, 0 RPM)	CD (mg/L)
1	0.30	100	2.00	2000	0.20	0.12	0.18
2	0.90	300	2.00	2000	0.34	0.16	0.74
3	1.50	500	2.00	2000	0.45	0.19	1.31
4	2.40	800	2.00	2000	0.57	0.23	2.17
5	3.00	1000	2.00	2000	0.79	0.50	2.50
6	3.60	1200	2.00	2000	1.38	1.22	2.38
7	4.20	1400	2.00	2000	1.74	0.53	3.67
8	5.10	1700	2.00	2000	2.69	1.40	3.70
9	6.00	2000	2.00	2000	3.28	2.07	3.93
10	6.90	2300	2.00	2000	3.93	2.84	4.06

**Table 11:** Dosage and residual chlorine levels for sodium hypochlorite and chitosan (L-ascorbic acid) during jar test at 2556 NTU raw water.

Jar	SH (mg/L)	SH (µL)	Chitosan (mg/L)	Chitosan (µL)	RC After Flocculation (30 min, 30 RPM)	RC After Sedimentation (178 min, 0 RPM)	CD (mg/L)
1	0.30	100	2.50	2500	0.09	0.06	0.24
2	0.90	300	2.50	2500	0.05	0.05	0.85
3	1.50	500	2.50	2500	0.11	0.06	1.44
4	2.40	800	2.50	2500	0.18	0.11	2.29
5	3.00	1000	2.50	2500	0.14	0.10	2.90
6	3.60	1200	2.50	2500	0.58	0.10	3.50
7	4.20	1400	2.50	2500	0.77	0.70	3.50
8	5.10	1700	2.50	2500	0.79	0.55	4.65
9	6.00	2000	2.50	2500	1.51	1.20	4.80
10	6.90	2300	2.50	2500	1.92	1.65	5.25

**Table 12:** Comparative Data of sodium Hypochlorite(SH) Dosage and Residual Chlorine.

	236.0 NTU RW		2556 NTU RW	
Acid Solution	SH Dose (mg/L)	RC (mg/L)	SH Dose (mg/L)	RC (mg/L)
Acetic acid	3.60	1.33	4.20	1.30
Citric acid	3.60	1.31	4.20	1.02
Hydrochloric acid	3.60	0.31	3.60	0.40
L-ascorbic acid	3.60	1.22	4.20	0.70
SH only (control)	4.20	2.09	4.20	1.95

**Table 13:** Comparative summary of dosage and cost for chitosan and conventional water treatment chemicals.

Treatment Type	Typical Dosage	Estimated Cost per 1,000,000 L	Chlorine Demand Reduction	Additional Benefits
**Sodium Hypochlorite (SH) Only**	4.20 mg/L	~$140 (at $0.48/lb for 7000 lb/day)	None	Disinfection only; high DBP potential
**Chitosan (as primary coagulant) [31]**	10–40 mg/L (depending on turbidity)	$100–$200 (at $10–$20/kg)	Moderate to High	Coagulation + Antimicrobial; reduces DBPs
**Chitosan (as additive)**	2–3 mg/L	$20–$60	High	Enhances existing coagulants; lowers chlorine demand
**Alum (Aluminum Sulfate)**	10–50 mg/L	~$60–$80	None	Requires pH adjustment; potential residuals

## Data Availability

The datasets generated during this study are available from the corresponding author upon request.
